# Morphologically intact airways in lung fibrosis have an abnormal proteome

**DOI:** 10.1186/s12931-023-02400-x

**Published:** 2023-04-01

**Authors:** Jeremy A. Herrera, Lewis A. Dingle, M. Angeles Monetero, Rajamiyer V. Venkateswaran, John F. Blaikley, Felice Granato, Stella Pearson, Craig Lawless, David J. Thornton

**Affiliations:** 1 The Wellcome Centre for Cell-Matrix Research, University of Manchester, Manchester Academic Health Science Centre, Manchester, Great Manchester UK; 2 Lydia Becker Institute of Immunology and Inflammation, University of Manchester, Manchester Academic Health Science Centre, Manchester, Great Manchester UK; 3 Blond McIndoe Laboratories, University of Manchester, Manchester Academic Health Science Centre, Manchester, Great Manchester UK; 4 Faculty of Biology, Medicine and Health, University of Manchester, Manchester Academic Health Science Centre, Manchester, Great Manchester UK; 5 Manchester University NHS Foundation Trust, Manchester, Greater Manchester UK; 6 Department of Medicine, University of Colorado Anschutz Medical Campus, Aurora, CO 80045 USA

## Abstract

Honeycombing is a histological pattern consistent with Usual Interstitial Pneumonia (UIP). Honeycombing refers to cystic airways located at sites of dense fibrosis with marked mucus accumulation. Utilizing laser capture microdissection coupled mass spectrometry (LCM-MS), we interrogated the fibrotic honeycomb airway cells and fibrotic uninvolved airway cells (distant from honeycomb airways and morphologically intact) in specimens from 10 patients with UIP. Non-fibrotic airway cell specimens from 6 patients served as controls. Furthermore, we performed LCM-MS on the mucus plugs found in 6 patients with UIP and 6 patients with mucinous adenocarcinoma. The mass spectrometry data were subject to both qualitative and quantitative analysis and validated by immunohistochemistry. Surprisingly, fibrotic uninvolved airway cells share a similar protein profile to honeycomb airway cells, showing deregulation of the slit and roundabout receptor (Slit and Robo) pathway as the strongest category. We find that (BPI) fold-containing family B member 1 (BPIFB1) is the most significantly increased secretome-associated protein in UIP, whereas Mucin-5AC (MUC5AC) is the most significantly increased in mucinous adenocarcinoma. We conclude that fibrotic uninvolved airway cells share pathological features with fibrotic honeycomb airway cells. In addition, fibrotic honeycomb airway cells are enriched in mucin biogenesis proteins with a marked derangement in proteins essential for ciliogenesis. This unbiased spatial proteomic approach generates novel and testable hypotheses to decipher fibrosis progression.

**Supplementary Information:**

The online version contains supplementary material available at 10.1186/s12931-023-02400-x.

## Introduction

Usual Interstitial Pneumonia (UIP) is a fibrotic disease that is associated with a variety of fibrotic entities (idiopathic pulmonary fibrosis—IPF, connective tissue disease—CTD, and hypersensitivity pneumonitis—HP) [1–3]. The UIP histological pattern is patchy with regions of relatively normal-appearing lung adjacent to dense fibrosis and honeycombing. Honeycomb refers to the clustering of airspaces within dense fibrotic tissue and is associated with the thickening of airway walls. Accumulation and plugging of mucus and other airway debris within the honeycomb airways impacts lung function.

Our current understanding of fibrotic airway pathogenesis has been improved with the advancement of structural, genetic, and molecular analyses. Structurally, UIP/IPF lung experiences reduced numbers of terminal bronchioles in regions of both minimal and established fibrosis [4–6]. Genetically, sequence changes affecting alveolar cells (*MUC5B*, *SFTPC*, and *SFTPA2*) have been reported [7–9]. Cells comprising the honeycomb airways present as either multi-layer or as a single layer [1], with cellular subtypes including; basal, ciliated, columnar, pseudostratified and secretory epithelium; while there are variable reports on the presence of alveolar type II (ATII) cells [10–12]. Functionally, single-cell RNA sequencing of IPF epithelial cells identify marked cellular heterogeneity as compared to control [13]. Collectively, these factors are believed to lead to airway homeostasis impairment and facilitate disease progression.

An important function of the airway is to produce mucus. Not only does mucus serve as a physical barrier but mucus also has antimicrobial properties to protect distal airways [14]. During the fibrotic process, mucus fills and plugs the honeycomb airways, which affects pathogen clearance and blood-oxygen exchange. The secreted mucus hydrogel is underpinned by two gel-forming mucins, of which, mucin 5B (MUC5B) is the most abundant in health, whereas MUC5AC is also detected but at a lower level [10]. A gain-of-function *MUC5B* polymorphism is amongst one of the highest risk factors associated with lung fibrosis [10, 15–18]. However, knowledge regarding the molecular composition of the mucus plug in UIP is incomplete.

We have recently created a tissue atlas of the fibrotic front of UIP/IPF utilizing laser capture microdissection coupled mass spectrometry (LCM-MS) to interrogate the fibrotic alveoli, fibroblastic foci and mature scar tissue [19, 20]. Here, we used the same approach to define the composition and provide mechanistic themes of the honeycomb airway cells with the aim to identify key targets and pathways to intercept fibrosis progression. In addition, we identify the composition of mucus in honeycomb airways in lung fibrosis (UIP) and compare this to lung cancer (mucinous adenocarcinoma) to determine if mucus heterogeneity exists in another progressive disease state where mucus plugs are found in the airways.

## Materials and methods

### Patient samples

Usual Interstitial Pneumonia (UIP) specimens were defined by current guidelines [1, 21]. Non-fibrotic controls were collected from morphologically normal lung tissue distal to tumor during resection (fibrotic and control patient demographics may be found in Additional file 1: Fig. S1). Mucinous adenocarcinoma was defined by current guidelines (mucinous adenocarcinoma patient demographics may be found in Additional file 1: Fig. S2) [22]. In this study, we utilized 10 UIP patients, 6 non-fibrotic patients, and 6 mucinous adenocarcinoma patients.

### Histological staining

Five-micron sections of formalin-fixed and paraffin-embedded (FFPE) specimens were H&E-stained by using an automated stainer (Leica XL) at the University of Manchester Histology Core Facility as previously described [19]. Importantly, slides were stored at 4 °C for up to one week while laser capture microdissection (LCM) was being performed. Captured material was stored at − 80 °C until all samples were ready for mass spectrometry processing. Alcian Blue/Periodic Acid Schiff (AB/PAS) was performed as follows. De-paraffinized slide sections were incubated for 5 min in 1% alcian blue 8GX (Sigma; A5268), 3% acetic acid. Slides were then washed in tap water followed by a 5-min incubation in 1% periodic acid (Sigma; 375810). Finally, slides were washed in tap water and incubated in Schiff’s reagent (Sigma—3952016) for 15 min. After extensive washing in tap water, slides were coverslipped without counterstain. For pentachrome, we followed a protocol as previously described [19].

For immunohistochemistry (IHC), we utilized the Novolink Polymer Detection Systems (Leica, RE7200-CE) as previously described [23]. We used the following antibodies anti-BPIFB1 (Abcam; ab219098, titre 1:60,000), anti-elastin (Proteintech; 15257-1-AP; titre 1:16,000), anti-PIGR (Abcam; ab224086, titre1:8000), and anti-serotransferrin (Abcam; ab268117, titre 1: 30,000). Anti-MUC5B (titre 1:10,000) and anti-MUC5AC (titre 1:12,000) was previously purified and used here [24]. For all samples, we used antigen heat retrieval using citrate buffer pH 6.0 (Sigma, C9999), with the exception of EDTA pH 9.0 antigen heat retrieval for serotransferrin and elastin. Slides were hematoxylin counterstained and coverslipped using permount (ThermoScientific, SP15).

For MUC5B, immunostains followed a modified protocol. After citrate buffer pH 6.0 antigen heat retrieval, the sections underwent reduction and alkylation. Sections were reduced by incubation at 37 °C for 30 min in 10 mM DTT, 0.1 M Tris/HCl pH 8.0. Sections were washed in water and then incubated in 25 mM Iodoacetamide, and 0.1 M Tris/HCl pH 8.0 for 30 min at room temperature (kept in the dark). Lastly, sections were washed in water followed by blocking and primary antibody incubation.

For immunofluorescence, dewaxed slides were subjected to citrate buffer pH 6.0 antigen heat retrieval and probed overnight with anti-MUC5AC (titre 1:100), anti-MUC5B (post reduction/alkylation; titre 1:100), or BPIFB1 (Abcam; ab219098, titre 1:100). Sections were then incubated with secondary anti-mouse fluorophore 680 (Invitrogen, A21058, 1:500) or anti-rabbit fluorophore 680 (Invitrogen; A21109; 1:500) for 1 h. Sections were coverslipped using ProLong antifade with DAPI (Invitrogen; P36931).

### Laser capture microdissection

The MMI CellCut Laser Microdissection System (Molecular Machines & Industries) was used to capture regions of interest on MMI membrane slides (MMI, 50102) as previously described [19, 20]. For this set of experiments, we collected a volume 0.03 mm^3^ of tissue per sample.

### Histological imaging

For fluorescence microscopy, all stains were performed at the same time. In addition, images were taken at the same intensity utilizing EVOS FL imaging system (ThermoScientific). For light microscopy, we used a DMC2900 Leica instrument with Leica Application Suite X software.

### Mass spectrometry sample preparation

Samples were prepared as described [19, 20]. In short, samples underwent a series of steps to maximize protein yield, including high detergent treatment, heating, and physical disruption.

### Liquid chromatography coupled tandem mass spectrometry

The separation was performed on a Thermo RSLC system (ThermoFisher) consisting of a NCP3200RS nano pump, WPS3000TPS autosampler and TCC3000RS column oven configured with buffer A as 0.1% formic acid in water and buffer B as 0.1% formic acid in acetonitrile. An injection volume of 4 µl was loaded into the end of a 5 µl loop and reversed flushed on to the analytical column (Waters nanoEase M/Z Peptide CSH C18 Column, 130 Å, 1.7 µm, 75 µm X 250 mm) kept at 35 °C at a flow rate of 300 ηl/min with an initial pulse of 500 ηl/min for 0.1 min to rapidly re-pressurize the column. The separation consisted of a multistage gradient of 1% B to 6% B over 2 min, 6% B to 18% B over 44 min, 18% B to 29% B over 7 min and 29% B to 65% B over 1 min before washing for 4 min at 65% B and dropping down to 2% B in 1 min. The complete method time was 85 min.

The analytical column was connected to a Thermo Exploris 480 mass spectrometry system via a Thermo nanospray Flex Ion source via a 20 µm ID fused silica capillary. The capillary was connected to a fused silica spray tip with an outer diameter of 360 µm, an inner diameter of 20 µm, a tip orifice of 10 µm and a length of 63.5 mm (New Objective Silica Tip FS360-20-10-N-20–6.35CT) via a butt-to-butt connection in a steel union using a custom-made gold frit (Agar Scientific AGG2440A) to provide the electrical connection. The nanospray voltage was set at 1900 V and the ion transfer tube temperature set to 275 °C.

Data was acquired in a data dependent manner using a fixed cycle time of 1.5 s, an expected peak width of 15 s and a default charge state of 2. Full MS data was acquired in positive mode over a scan range of 300 to 1750 Th, with a resolution of 120,000, a normalized AGC target of 300% and a max fill time of 25 mS for a single microscan. Fragmentation data was obtained from signals with a charge state of + 2 or + 3 and an intensity over 5000 and they were dynamically excluded from further analysis for a period of 15 s after a single acquisition within a 10-ppm window. Fragmentation spectra were acquired with a resolution of 15,000 with a normalized collision energy of 30%, a normalized AGC target of 300%, first mass of 110 Th and a max fill time of 25 mS for a single microscan. All data was collected in profile mode.

### Mass spectrometry data analysis and statistics

Raw data for regional airway cell samples were processed using MaxQuant [25] version 1.6.17.0 against the human proteome obtained from uniprot (May 2021) [26]. Raw data for UIP and mucinous adenocarcinoma mucus samples were processed using MaxQuant [25] version 2.0.3.0 against the human proteome obtained from uniprot (May 2022) [26]. All Maxquant processing were performed with a fixed modification of carbamidomethylation of cysteine, with variable modifications of methionine oxidation and protein N-terminal acetylation. Precursor tolerance was set at 20 ppm and 4.5 pm for the first and main searches, with MS/MS tolerance set at 20 ppm. A false discovery rate (FDR) of 0.01 was set for PSM and protein level, up to two missed cleavages were permitted and “match-between-runs” was selected.

Stastical analysis was carried out in R (v4.1.2) [27] using the MSqRob package (v0.7.7) [28]. Significantly changing proteins were taken at a 5% false discovery rate (FDR). Pathway analysis utilising Reactome Pathways was performed on significantly changing proteins using the R package ReactomePA (1.38.0) [29].

## Results

### Laser capture microdissection of fibrotic and non-fibrotic airway cells

**Fig. 1:**
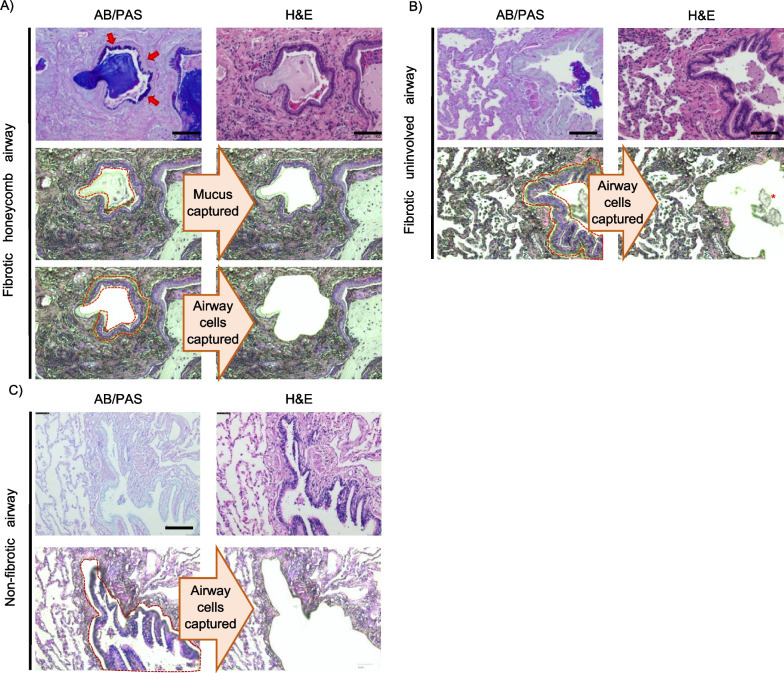
Laser capture microdissection of the mucus, fibrotic honeycomb, fibrotic uninvolved and non-fibrotic airway cell controls. Formalin-fixed paraffin-embedded specimens were serially sectioned at 5 microns and stained with alcian blue/periodic acid Schiff’s (AB/PAS) stain or Hematoxylin & Eosin (H&E). **A** A representative image of laser capture microdissection in one fibrotic specimen. AB/PAS (Top left) or H&E (the other 5 panels). Mucus (purple) was visualized with AB/PAS stain; notice how the mucin lines the inner airway consistent with these cells producing mucin centrally into the airspace [red arrows]. We individually captured the mucus and fibrotic honeycomb airway cells for mass spectrometry preparation. **B** In the same fibrotic patient, we found uninvolved airways in the morphologically intact regions of the fibrotic lung and captured the airway cells for mass spectrometry preparation (notice how the mucus plug is left behind depicted with red asterisk). **C** A representative image of laser capture microdissection of a non-fibrotic airway control captured for mass spectrometry preparation. Scale bar represents 100 microns

Figure 1 shows our approach to laser capture microdissection (LCM). Using alcian blue/ periodic acid Schiff’s (AB/PAS) stain, mucus is visualized as purple in color within the fibrotic honeycomb airway (Fig. 1A, upper row). Note how the AB/PAS stain lines the airway cells (red arrows) in a manner that suggests mucin is being secreted centrally into the airway lumen. We show that we precisely captured the mucus in a fibrotic specimen, including its cellular infiltrates (Fig. 1A, middle row). In addition, we captured the mucin-rich epithelial lining of honeycomb airways (Fig. 1A, lowest row) and the fibrotic uninvolved airway cells defined as being in distant regions demonstrating minimal fibrosis (Fig. 1B). Our LCM capabilities allow us to precisely isolate this region while leaving behind mucus associated in uninvolved airways, denoted with a red asterisk. To serve as a control, we performed LCM on airway cells from non-fibrotic control specimens (Fig. 1C). In total, we performed LCM on 10 fibrotic specimens (n = 10 fibrotic honeycomb airway cells, n = 10 fibrotic uninvolved airway cells) and on 6 non-fibrotic airway control cells (a total of 26 samples and a total of 16 patients).

### The fibrotic honeycomb and uninvolved airway cells are similar in protein composition

**Fig. 2:**
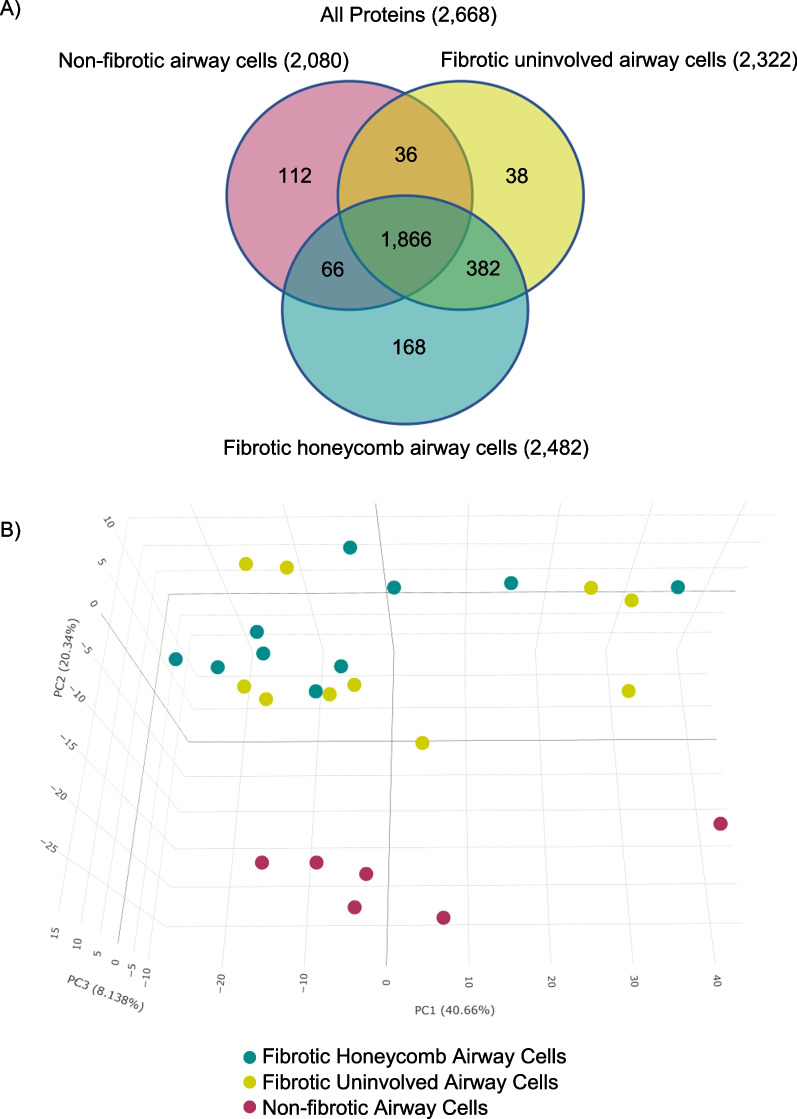
Spatial proteomic analysis of the fibrotic airway cells. Fibrotic and non-fibrotic control specimens were subjected to laser capture microdissection coupled mass spectrometry (LCM-MS) to collect non-fibrotic airway cells (n = 6 control patients), fibrotic uninvolved airway cells (n = 10 UIP patients), and fibrotic honeycomb airway cells (n = 10 UIP patients). **A** Venn diagram showing the number of proteins found in each airway cell type. **B** 3-D Principal component analysis showing that the non-fibrotic airway cells (red dots) cluster away from the fibrotic airway cells (the other dots). Surprisingly, fibrotic uninvolved airway cells (yellow dots) and honeycomb airway cells (green dots) cluster together

We prepared our samples for mass spectrometry (MS) following our established protocol [19, 20] and performed a qualitative analysis to determine which proteins were present per group: non-fibrotic control (n = 6 patients), fibrotic uninvolved (n = 10 UIP patients), and fibrotic honeycomb airway cells (n = 10 UIP patients). We define a protein present if it is detected in 3 of the 6 non-fibrotic airway cell samples or 5 of the 10 fibrotic airway cell samples. We detected 2668 proteins in human lung airway cells (Fig. 2A) and provide a complete list of these proteins (Additional file 2). We found that more proteins are detected in fibrotic honeycomb airway cells, which may be attributed to the metabolically demanding process of mucin production [30].

We next performed a quantitative analysis, which compared the relative abundance of the detected proteins, to create a 3-dimensional (3-D) principal component analysis (PCA) (Fig. 2B). Firstly, we showed that non-fibrotic airway cells (red dots) separate from both the fibrotic honeycomb (green dots) and fibrotic uninvolved airway cells (yellow dots). Surprisingly, we found that both the fibrotic honeycomb airway cells and fibrotic uninvolved airway cells closely cluster with some deviation. This analysis suggests that fibrotic uninvolved airway cells (found in morphologically intact lung) display an abnormal protein profile similar to the mucin-rich honeycomb airway cells (found within the densely fibrotic region of the lung).

### Honeycomb airway cells are enriched in proteins involved in mucin biogenesis and have decreased cilia-associated proteins

**Fig. 3:**
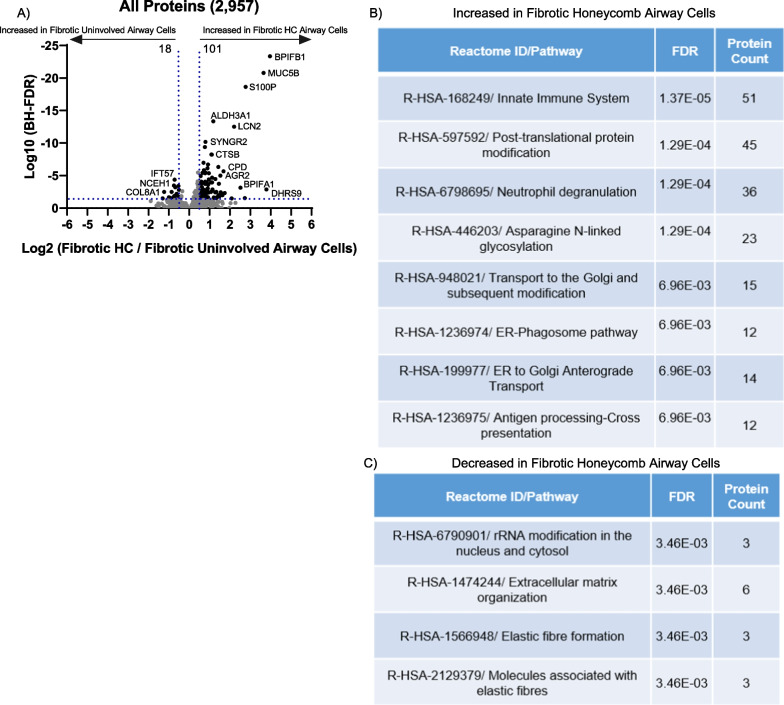
The fibrotic honeycomb airway cells have a pro-mucin protein signature. **A** Volcano plot comparing the fibrotic honeycomb [HC] airway (n = 10 UIP patients) to fibrotic uninvolved airway cells (n = 10 UIP patients) showing the negative natural log of the false discovery values (FDR) values plotted against the base 2 log (fold change) for each protein. Reactome pathway showing the most **B** increased or **C** decreased for the fibrotic honeycomb airway cells compared to fibrotic uninvolved airway cells

Although the fibrotic honeycomb and fibrotic uninvolved airway cells cluster by PCA, we sought to further compare these groups. Of the 2,957 proteins detected, we found that there are 101 proteins significantly increased in fibrotic honeycomb airway cells while 18 are statistically increased in the fibrotic uninvolved airway cells (Fig. 3A, a full list in Additional file 3). A list of the highest and lowest proteins is provided in Table 1. Consistent with our approach of capturing mucin-rich honeycomb airway cells, we found that MUC5B is significantly increased in the fibrotic honeycomb airway cells and not in the fibrotic uninvolved airway cells.

**Table 1:** Highest and lowest 15 significantly changed proteins in the fibrotic honeycomb airway cells compared to fibrotic uninvolved airway cells

Increased in fibrotic honeycomb airway cells			Decreased in fibrotic honeycomb airway cells		
Protein	Log_2_	FDR	Protein	Log_2_	FDR
BPIFB1	3.97	4.60E−24	CEP135	− 1.30	3.18E−02
DHRS9	3.79	1.40E−03	COL8A1	− 1.23	3.28E−03
MUC5B	3.65	1.70E−21	FBLN2	− 1.00	2.16E−02
S100P	2.77	2.40E−19	LRRC45	− 0.90	2.88E−02
FAM3D	2.73	2.90E−02	TTC21B	− 0.86	3.29E−03
BPIFA1	2.52	7.40E−04	LAMB2	− 0.75	3.40E−04
LCN2	2.20	3.13E−13	APCS	− 0.74	4.45E−02
CRABP2	2.08	3.22E−02	CGN	− 0.74	1.45E−02
DMBT1	1.74	4.82E−03	FBN1	− 0.72	2.24E−02
CPD	1.68	2.30E−06	IFT57	− 0.72	4.30E−05
ST6GAL1	1.64	1.02E−02	NCEH1	− 0.69	6.30E−04
IGJ	1.55	3.763E−03	PLG	− 0.63	8.00E−03
AGR2	1.53	1.00E−05	H1F0	− 0.62	6.25E−03
FKBP11	1.49	1.53E−02	CYP51A1	− 0.60	2.90E−02
SLPI	1.46	1.80E−04	CERS2	− 0.59	2.59E−02

Strikingly, many of the proteins increased in the fibrotic honeycomb airway cells are involved in mucin biogenesis and/or regulation. For instance, bactericidal/permeability-increasing (BPI) fold-containing family B member 1 (*BPIFB1*) is a negative regulator of *MUC5B* expression [31] and is at the top of the list. Similarly, secretory leukocyte protease inhibitor (*SLPI*) reduces mucin expression in vitro and is enriched in the fibrotic honeycomb airway cells [32]. This suggests that negative regulators of mucin expression in lung fibrosis are insufficient to stop mucin production. In accord, Reactome pathway analysis demonstrated that a variety of pathways pertaining to mucin production are increased, such as ‘Post-translational protein modification’, ‘Transport to the Golgi and subsequent modification’, and ‘ER-phagosome pathway’ (Fig. 3B); however, it is noteworthy that mucin biogenesis is not an established Reactome pathway.

Reactome pathway analysis also demonstrates that ‘extracellular matrix organization’ and ‘elastic fibre formation’ are decreased in the fibrotic honeycomb airway cells (Fig. 3C). We confirm that there are disorganized elastic fibres in the honeycomb airways, which is in accord with the loss of airway structure and increased fibrosis in this region (Additional file 1: Figure S3). Thus, spatial proteomics identifies a pro-mucin protein signature associated with the fibrotic honeycomb airway cells as compared to fibrotic uninvolved airway cells.

**Fig. 4:**
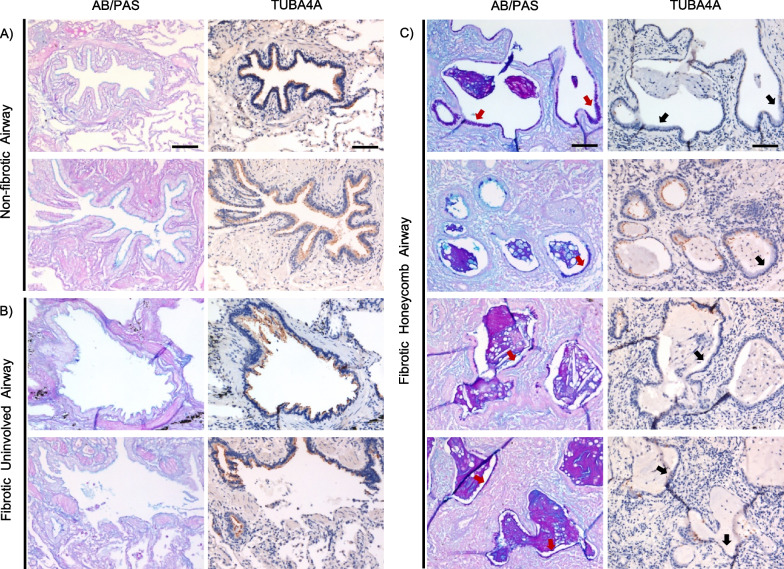
Cilia expression in the fibrotic honeycomb airway cells. Two non-fibrotic control and 4 fibrotic patient specimens were stained for alcian blue/periodic acid Schiff’s (AB/PAS) or immunostained for TUBA4A (a marker of cilia). Shown are representative images for **A** non-fibrotic control airway, **B** fibrotic uninvolved airways, and **C** fibrotic honeycomb airways. Note that regions of mucin positivity (red arrows) are absent of cilia (black arrows) in the fibrotic honeycomb airway cells. Scale bar represents 100 microns

Cilia are conserved organelles that function to clear airway mucus and associated debris. Herein, we demonstrated that multiple proteins associated with ciliogenesis are decreased in the fibrotic honeycomb airway cells. For instance, Centrosomal protein 135 (CEP135) is the most decreased protein and is required for ciliogenesis initiation [33]. To demonstrate that abnormal ciliogenesis is a potential mechanistic theme in the fibrotic honeycomb airway cells, we immunostained for tubulin alpha 4a (TUBA4*A*; a marker of cilia) in 4 UIP specimens and 2 controls. We found that the cilia marker is widely expressed in cells lining the airway cells of non-fibrotic and fibrotic uninvolved airways (Fig. 4A, B). In contrast, the mucin-rich regions of the honeycomb airway (red arrows) are largely devoid of cilia (black arrows) (Fig. 4C). Thus, our dataset confirms features of honeycombing offering a variety of known and new targets to understand fibrosis progression.

### Fibrotic uninvolved airway cells have an abnormal protein signature

**Fig. 5:**
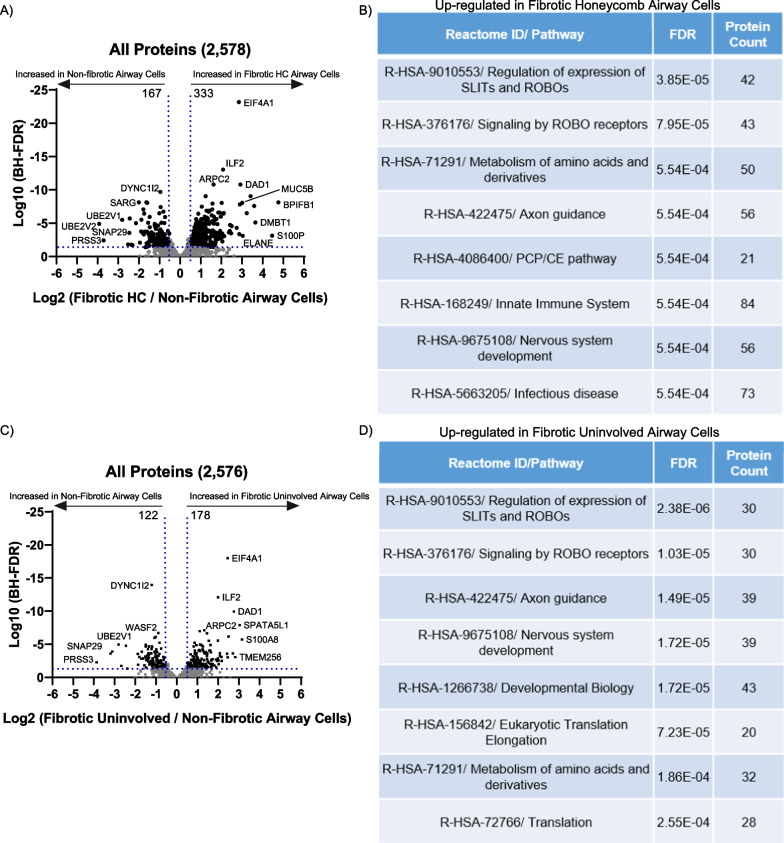
The fibrotic uninvolved airway cells have an abnormal protein signature. **A** Volcano plot comparing the fibrotic honeycomb [HC] airway cells (n = 10 UIP patients) to non-fibrotic airway cells (n = 6 control patients) showing the negative natural log of the false discovery values (FDR) values plotted against the base 2 log (fold change) for each protein. **B** Reactome pathway showing the most increased pathway for the fibrotic honeycomb airway cells compared to non-fibrotic airway cells. **C** Volcano plot comparing the fibrotic uninvolved airway cells (n = 10 UIP patients) to non-fibrotic airway cells (n = 6 control patients) showing the negative natural log of the FDR values plotted against the base 2 log for each protein. **D** Reactome pathway showing the most increased pathway for the fibrotic uninvolved airway cells compared to non-fibrotic airway cell controls

We next compared fibrotic airway cells (n = 10 UIP patients) to the non-fibrotic airway cells (n = 6 control patients). We showed that 333 proteins are significantly increased in the fibrotic honeycomb airway cells, whereas 157 proteins are significantly increased in the non-fibrotic airway cell controls (Fig. 5A; a full list in Additional file 3). Reactome pathway analysis demonstrated that ‘regulation of expression of *SLIT*s and *ROBO*s’ and ‘Signaling by *ROBO* receptors’ are the strongest categories increased in the fibrotic honeycomb airway cells as compared to non-fibrotic controls (Fig. 5B). The slit protein and their roundabout receptors (Slit and Robo) pathway is largely involved in cell migration [34].

We next compared the fibrotic uninvolved airway cells (n = 10 UIP patients) to the non-fibrotic airway cells (n = 6 control patients). We detected 178 proteins significantly increased in fibrotic uninvolved airway cells, whereas we found 202 proteins significantly increased in non-fibrotic airway cell controls (Fig. 5C; a full list in Additional file 3). Surprisingly, the 15 highest proteins increased in the fibrotic uninvolved airway cells are also increased in the fibrotic honeycomb airway cells. Reactome pathway analysis again show that ‘Regulation of expression of *SLIT*s and *ROBO*s’ and ‘Signaling by *ROBO* receptors’ are the strongest categories in the fibrotic uninvolved airway cells as compared to non-fibrotic controls (Fig. 5D); we do not detect any decreased Reactome pathways for either fibrotic airway cells as compared to control.

**Fig. 6:**
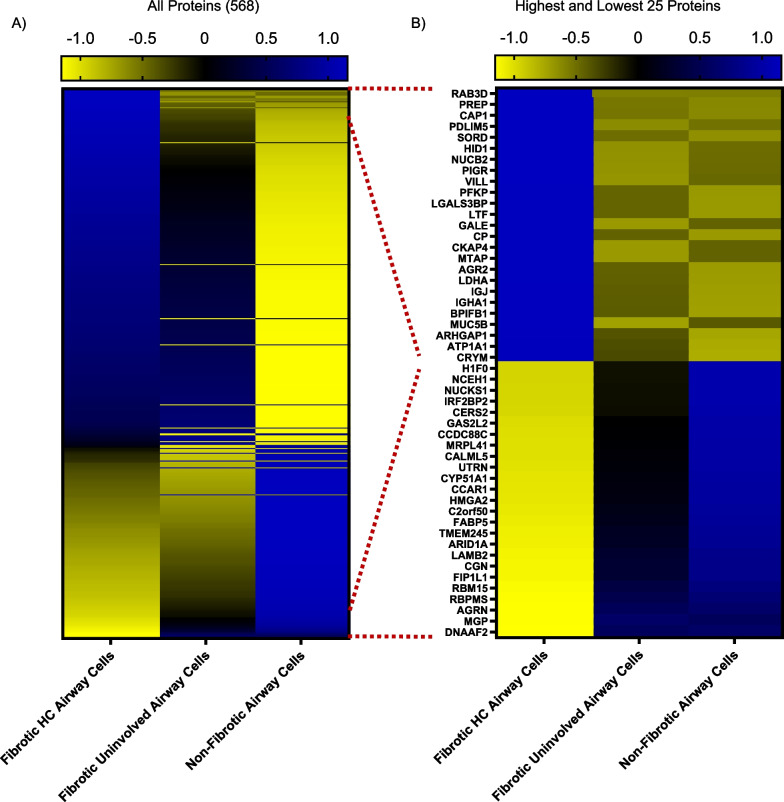
Fibrotic airway cells differ than controls. Shown are heatmaps of **A** all 568 statistically changed proteins or **B** the highest and lowest 25 proteins in the fibrotic honeycomb [HC] (n = 10 UIP patients), fibrotic uninvolved (n = 10 UIP patients), and non-fibrotic airway cells (n = 6 control patients). Proteins are arranged by increasing abundance with reference to the honeycomb airway cells

A heatmap of the 568 significantly changed proteins across the groups: fibrotic honeycomb, fibrotic uninvolved, and non-fibrotic airway cell controls is shown in Fig. 6A (a full list in Additional file 4). The fibrotic honeycomb airway cells share features with the fibrotic uninvolved airway cells. We next show the 25 highest and lowest changed proteins (Fig. 6B). Ras-related protein 3D (RAB3D) was the most increased in the fibrotic honeycomb airway cells and is involved in the biogenesis of secretory granules [35]. MUC5B and MUC5AC are both packaged in the secretory granules of airway cells [36]. Similarly, prolyl endopeptidase (PREP) is found within exosomes in airway cells and released upon LPS stimulation [37]. Cyclase associated protein 1 (CAP1) is associated with lung cancer and post-translational modification promotes proliferation and migration [38]. At the bottom of the list are dynein axonemal assembly factor 2 (DNAAF2), matrix gla protein (MGP), and agrin (AGRN) which are only decreased in the fibrotic honeycomb airway cells (detected in the fibrotic uninvolved and non-fibrotic airway cell controls). *DNAAF2* is involved in cilia homeostasis and mutations in *DNAAF2* lead to cilia defects [39]. *MGP* is considered an inhibitor of calcification based on the extensive cardiovascular calcification observed in *MGP*-null mice [40]; calcification occurs in UIP/IPF patients, and is associated within regions of honeycombing [41]. *AGRN* is a proteoglycan that serves a variety of biological functions, including the promotion of regeneration [42]. Further work interrogating the collective roles of these changed proteins might help decipher the mechanism of fibrosis progression. Given that similar pathways and proteins are increased in the fibrotic uninvolved airway cells as the fibrotic honeycomb airway cells, our data indicate that fibrotic uninvolved airway cells are abnormal.

### The composition of fibrotic lung mucus

**Fig. 7:**
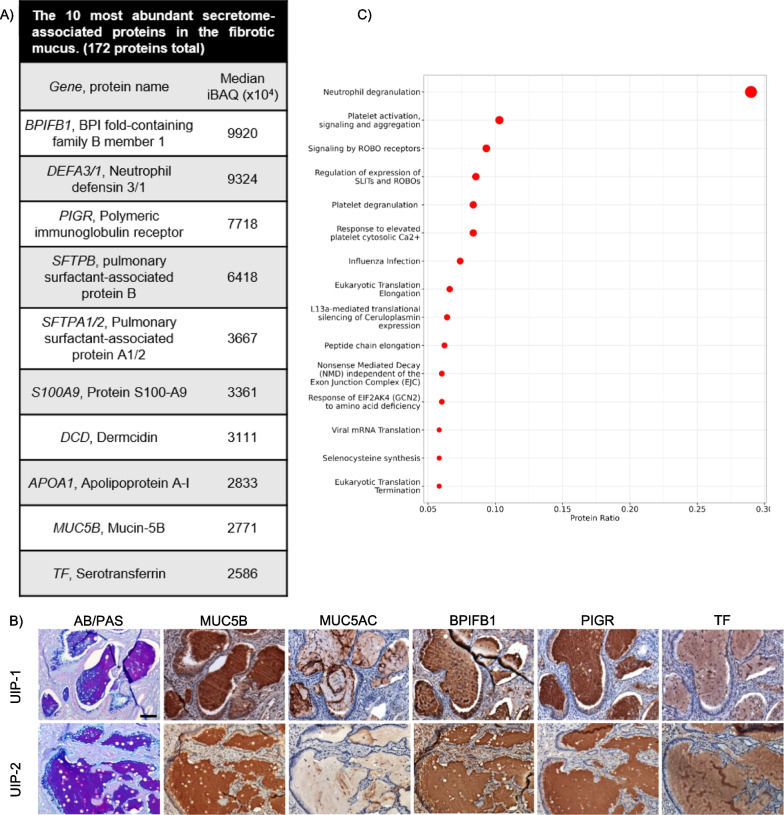
The composition of fibrotic mucus. Laser capture microdissection coupled mass spectrometry was performed on the mucus plugs of 6 Usual Interstitial Pneumonia (UIP) patients. **A** A list of the most abundant secretome-associated proteins found in the fibrotic mucus shown as intensity Based Absolute Quantification (iBAQ). **B** Reactome pathway enrichment of UIP mucus represented as a dotplot. **C** Serial sections of UIP specimens stained for alcian blue/periodic acid Schiff’s (AB/PAS) or immunostained for MUC5B, MUC5AC, BPIFB1, PIGR, and TF (N = 4 UIP patients with 2 representative images shown). Scale bar represents 100 microns

Utilizing our MS approach, we detected 650 proteins in the fibrotic/UIP mucus plugs (detected in 3 or more of the 6 samples; Additional file 5). Using intensity Based Absolute Quantification (iBAQ; a measure of protein abundance) [43], we provide a list of the most abundant proteins in UIP mucus (Additional file 6). We found that the mucus is enriched with immunoglobulins (Ig) which is in accord with increased protein expression of polymeric Ig receptor (PIGR) in the fibrotic honeycomb airway cells and mucus. In epithelial cells, PIGR mediates the transcytosis of Igs into the airway, which serves as a mucosal defence mechanism [44]. Given that fibrotic mucus is enriched with cellular infiltrates, we next focused our list using the ‘secretome’ (secreted proteins) dataset [45] and show that BPIFB1 was the most abundant secretome-associated protein found in fibrotic mucus whereas MUC5B is the ninth most abundant (Fig. 7A; a full list in Additional file 7). This is consistent with BPIFB1 and MUC5B being amongst the most significantly expressed proteins in the fibrotic honeycomb airway cells (Fig. 3A). To validate some the most abundant protein hits, we show immunoreactivity for MUC5B, BPIFB1, PIGR, and TF within the UIP mucus plugs (Fig. 7B). We also included the other gel-forming mucin, MUC5AC (60th on the abundance list), which showed a patchy/incomplete staining pattern.

Using the entire fibrotic mucus proteome, Reactome pathway enrichment analysis demonstrates that the mucus plug is defined by ‘neutrophil degranulation’ as the strongest category (Fig. 7C). The neutrophil degranulation pathway is also implicated in SARS-CoV-2 lung infection models [46] and in chronic obstructive pulmonary disease (COPD) [47]; in IPF bronchoalveolar lavage fluid (BALF), proteins associated with neutrophil granules are amongst the most abundant [48].

### Fibrotic-derived lung mucus is distinct from cancer-derived lung mucus

Due to the absence of mucus in non-fibrotic airway controls, we sought to further understand mucus in the context of another lung disease. We performed LCM-MS on 6 mucinous adenocarcinoma specimens (Additional file 1: Fig. 4A). mucinous adenocarcinoma is a lung cancer with pronounced mucus accumulation within the alveolar space [22, 49]. We detected a total of 535 proteins in mucinous adenocarcinoma mucus (found in 3 or more of the 6 samples; a full list in Additional file 5). Consistent with *MUC5AC* being the most abundantly expressed transcript in mucinous adenocarcinoma [50], MUC5AC protein is the second most abundant secretome-associated protein found in the mucus of mucinous adenocarcinoma (Additional file 1: Fig. 4B, a full list in Additional file 6). Reactome pathway analysis demonstrates that mucinous adenocarcinoma mucus is defined by ‘Neutrophil degranulation’, like fibrotic mucus, as the strongest category (Additional file 1: Fig. 4C).

**Fig. 8:**
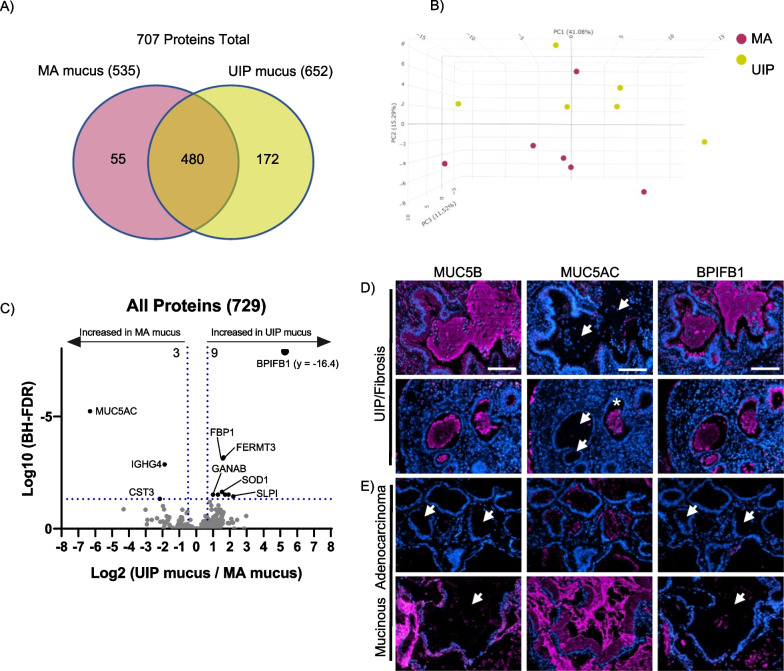
The mucus in usual interstitial pneumonia has distinct features from mucinous adenocarcinoma. **A** Venn diagram showing the proteins detected in the mucus of mucinous adenocarcinoma [MA] (n = 6 patients) or usual interstitial pneumonia (UIP; n = 6 patients). **B** 3-dimensional principal component analysis for each mucus type. **C** Volcano plot comparing the UIP mucus to mucinous adenocarcinoma mucus showing the negative natural log of the false discovery values (FDR) values plotted against the base 2 log (fold change) for each protein. **D** Immunofluorescence for MUC5B, MUC5AC, and BPIFB1 in UIP mucus (n = 4 patients) and mucinous adenocarcinoma mucus (n = 3 patients) with representative images shown for each disease type. White arrows points to regions of mucus accumulation and asterisk shows positivity of MUC5AC within UIP mucus. Scale bar represents 100 microns

We next compared the mucus of mucinous adenocarcinoma to UIP. In total, we detected 707 lung mucus proteins (Fig. 8A), with UIP having the most proteins detected (a full list in Additional file 5). A 3-D PCA analysis showed that UIP mucus samples largely cluster together, whereas only one mucinous adenocarcinoma sample overlaps with UIP (Fig. 8B). Quantitative analysis of our data show that 9 proteins are significantly enriched in fibrotic mucus whereas 3 are significantly enriched in mucinous adenocarcinoma mucus (Fig. 8C, a full list in Additional file 8). To validate this result, we performed immunofluorescence on both mucinous adenocarcinoma (n = 3) and UIP specimens (n = 4) for MUC5B, MUC5AC, and BPIFB1 (Fig. 8D). We found that UIP mucus has variable expression of MUC5AC (white arrows mark the absence of MUC5AC where MUC5B/BPIFB1 is present) in comparison to MUC5B. Note that one mucus plug was positive for MUC5AC, which resembles the chromogenic patchy/incomplete stain in Fig. 7B; MUC5AC has been previously reported to have variable staining [10]. Inversely, mucinous adenocarcinoma mucus was positive for MUC5AC, whereas MUC5B and BPIFB1 are largely negative at the immunofluorescence level (Fig. 8E, white arrows point where MUC5B/BPIFB1 are absent). Thus, a distinguishing factor for fibrotic/UIP mucus is the high abundance of MUC5B and BPIFB1, whereas MUC5AC is predominant in mucinous adenocarcinoma mucus.

## Discussion

**Fig. 9:**
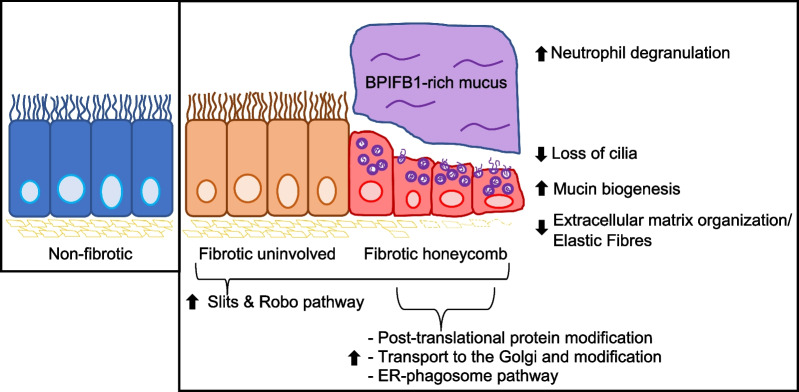
The fibrotic honeycomb airway. Spatial proteomics reveal that the fibrotic uninvolved airway cells (found in regions of structurally intact lung) have an abnormal protein signature. The fibrotic uninvolved airway cells, like the honeycomb airway cells (a pathological feature of lung fibrosis), are over-represented in proteins associated with slits and Roundabout protein (Slit and Robo) pathway. The fibrotic honeycomb airway cells are further defined by increased pathways associated with mucin biogenesis, and a loss of both cilia and ECM organization/elastic fibres. We find that the mucus proteome is enriched with neutrophil degranulation pathway, with a marked increase of BPIFB1 protein

In this work, we aimed to create a protein tissue atlas of airway cells to understand fibrosis progression. We produced an unbiased spatial proteomic profile of the non-fibrotic, fibrotic uninvolved and honeycomb airway cells to highlight pathways that may intercept fibrosis progression (Fig. 9). We showed that the structurally intact low-in-mucus airway cells in uninvolved regions of the fibrotic lung share an abnormal protein signature with fibrotic honeycomb airway cells featuring increased Slit and Robo pathway as the strongest category. Given that Slit and Robo pathway is primarily implicated in cell migration, lung injury and development [34, 51–53], we speculate that Slit and Robo may be part of the mechanism of fibrosis initiation. This is supported with the recent finding that IPF airway epithelium have an increased migratory phenotype [54]. Thus, the data here will be the premise of future studies interrogating the mechanistic impact of Slit and Robo pathway in lung fibrosis.

In accord with our current understanding of lung fibrosis, this unbiased approach confirms that the fibrotic honeycomb airways are the site of mucin biogenesis with other categories related to protein modification and transport increased. For instance, retinoic acid signalling induces mucin gene expression and secretion [55, 56]; Dehydrogenase reductase SDR family member 9 (*DHRS9*) and cellular retinoic acid-binding protein 2 (*CRABP2*) both modulate retinoic acid synthesis and are increased in the fibrotic honeycomb airway cells. Recently, CRABP2 was shown to be increased in IPF airway cells [57]. In accord with mucin biogenesis, anterior gradient homolog 2 (*AGR2*) has been shown to be essential for MUC2 production and FK506-binding protein 11 (*FKBP11*) has been demonstrated to have a mucin secretory function [58]. Both AGR2 and FKBP11 are increased in the fibrotic honeycomb airway cells. Some unique proteins to the fibrotic honeycomb airway cells include GALNT12, GALNT3, ST6GAL1, and GALNT6, which are associated with O-linked glycosylation of mucins. Although a subset of these results are confirmatory to airway derangements in lung fibrosis, this rich dataset leaves readers with many novel proteins for which their functions in fibrosis progression are unknown.

Aberrant ciliogenesis has previously been described in UIP/IPF. Whole transcriptomic studies demonstrate an elevation of cilium gene expression [59]. In contrast, our results demonstrate a reduced cilia-associated protein profile within the fibrotic honeycomb airway cells. This likely reflects the advancement of our spatial proteomic capability. Ciliogenesis relies on a variety of proteins, including intraflagellar transport (IFT) proteins [60]. Intraflagellar transport protein 57 (IFT57) is required for cilia maintenance and is decreased in the fibrotic honeycomb airway cells; other intraflagellar transport proteins (IFT81, IFT46) are not expressed in the fibrotic honeycomb airway cells but expressed in the non-fibrotic or fibrotic uninvolved airway cells. Leucine-rich repeat protein 21B (*LRRC45*) and tetratricopeptide repeat protein 21B (*TTC21B*) are critical for ciliogenesis and are also decreased in the fibrotic honeycomb airway cells [61, 62]. A variety of proteins that are not expressed in the fibrotic honeycomb airway cells include proteins associated with cilia function, such as CEP131, CCP110, KIF3A, CYB5D1, DYNLRBR2, RPGR, and WRD66 [63–69]. In support of a deranged cilia phenotype, transmission electron microscopy demonstrates that the UIP/IPF distal airways display defects in microtubule organization [70]. In the context in cystic fibrosis, it is reported that airway epithelial also have decreased ciliated cells with enhanced mucin expression [71]. Thus, future studies determining the mechanism of deranged ciliogenesis is warranted.

Other groups support our results that demonstrate that the fibrotic uninvolved airway cells are abnormal at the structural and genetic level. Lung fibrosis is associated with a variety of genetic risk factors affecting epithelial cells, which may abnormally prime lung airway cells to fibrosis initiation [72]. Structurally, regions without microscopic fibrosis are shown to have reduced numbers of terminal airways and have an increase of airway wall areas [4–6], suggesting that early lung airway perturbations precede fibrotic extracellular matrix remodelling. Thus, it is plausible that airway cell dysfunction is an early event in the fibrotic process. Further LCM-MS studies with precise distance registration and patient genotyping will inform whether there exists a transition zone where a normal airway proteome is present, or perhaps it may be that the entire airway proteome is abnormal.

Current literature suggests that basal airways cells differentiate into either mucin producing cells or cilia-containing cells [73]. Our spatial proteomic data fits the notion that the honeycomb airway microenvironment directs the differentiation of basal airway cells into mucin producing cells whereas the uninvolved airway microenvironment favors ciliated cells. Given that extracellular matrix governs cell differentiation and function [74], we speculate that changes to extracellular matrix properties (mechanical, composition, and topography) within the honeycomb airway may play a role in airway cell differentiation and mucin biogenesis. Prior work utilizing decellularized COPD airway tissue as a scaffold for cell–matrix interactions (as compared to donor) show that COPD matrix dramatically affects cilia gene expression in epithelial cells [75]. Other studies using decellularized UIP/IPF tissue confirm that fibrotic matrix is a driver of fibrosis progression [76]. Therefore, the changes in mucin and cilia-associated proteins may be reflective, or a consequence of the changes in airway extracellular matrix properties.

Our spatial proteomic data characterizing fibrotic honeycomb airway cells (MUC5B-positive) are in agreement with sc-RNAseq data characterizing *MUC5B*-positive secretory cells in human lung. In one study, secretory airway cells have increased RNA expression of *MUC5B*, *LCN2*, *BPIFB1*, *SERPINB3*, *S100P*, *RARRES1*, *TSPAN8*, *CP*, and *FAM3D* [77], which are also increased or uniquely expressed at the protein level in fibrotic honeycomb airway cells. Other mRNAs increased in *MUC5B*-positive secretory cells include *TSPAN1*, *AKR1C1*, *ZG16B*, *GSTA1*, and *SCGB1A1,* which are unchanged at the protein level in the fibrotic honeycomb airway cells. A separate study showed that *MUC5B*-positive cells by sc-RNAseq have increased mRNA expression of *SCGB1A1*, *SCGB3A1*, *SLPI*, *BPIFB1*, *LCN2*, and *WFDC2* [78]. At the protein level, SLPI, BPIFB1, LCN2, and WFDC2 are increased or uniquely expressed in the fibrotic honeycomb airway cells (SCGB1A1 and SCGB3A1 are unchanged at the protein level). Thus, the fibrotic honeycomb airway cells represent a secretory cell phenotype. Future work integrating spatial multi-omic analysis (RNA and protein) will further our understanding of lung fibrosis.

To our knowledge, we are the first to determine the composition of UIP mucus plugs by using a LCM-MS approach. This approach allows precise capture of the entirety of mucus plugs without the introduction of contaminants (salivary and upper airway proteins) as seen by traditional BALF. Proteomic analysis of BALF (an unfixed or stained sample) from lung fibrosis patients show agreement with our findings. Several reports utilizing mass spectrometry approaches show increases of immunoglobulins, complement C3, transferrin, Apolipoprotein A1, plastin-2, annexin A2, and CCL18 in fibrotic lung BALF (summarized in [79]); all of which are detected in our LCM-MS dataset. In accord with our findings, Foster et al. demonstrated that MUC5B is an abundant protein in IPF BALF [48]. S100A9, detected by LCM-MS, is a potential BALF biomarker in IPF [80]. In addition, IPF patients with acute exacerbations show increased PIGR, LRG1, and SERPINA1 in BALF, which are also detected in our LCM-MS dataset [81]. Our LCM-MS approach is therefore a useful tool to determine the protein composition of mucus in archived formalin-fixed paraffin-embedded specimens.

Our results demonstrate that the mucus found in lung cancer (mucinous adenocarcinoma) has elevated levels of MUC5AC as compared to UIP mucus. A likely explanation is that the mucin-secreting cells comprising the UIP/IPF honeycomb airway differ than the mucin-secreting cells in mucinous adenocarcinoma and/or that the environmental/immune signals controlling mucin production differ. For instance, reports show that there are 5 times more MUC5B-positive cells versus MUC5AC-positive cells in the honeycomb airways of UIP/IPF, suggesting marked cell type heterogeneity [82]. In contrast, the morphology of mucinous adenocarcinoma cells are distinct and composed of goblet and/or columnar cells [83]. Another explanation is that *MUC5AC* gene expression is differentially regulated as compared to *MUC5B* [84]. For instance, *MUC5AC* gene expression is increased by IL-13. In other disease settings, *MUC5AC* mRNA is increased in asthma, whereas *MUC5B* levels are decreased [85]. Further studies determining the functional consequence of varying MUC5AC to MUC5B protein ratios on fibrosis progression are needed.

Increases of BPIFB1 in both the UIP mucus and honeycomb airway cells is of interest. BPIFB1 is a secretory protein that is implicated in immune regulatory functions and shown to have anti-tumor effects (reviewed in [86]). In other lung disorders, BPIFB1 is increased in cystic fibrosis, COPD, asthma, and IPF [87]. It is decreased in nasopharyngeal carcinoma, gastic cancer, and lung cancer, which agrees with our findings that mucinous adenocarcinoma mucus has low expression of BPIFB1. Understanding of its function in lung fibrosis is currently incomplete.

## Conclusion

Spatial proteomics has allowed us to create an unbiased protein tissue map of the fibrotic/UIP lung airway cells. We show that the fibrotic honeycomb airway cells are the active site of mucin biogenesis affiliated with a loss of cilia. Importantly, we show that the fibrotic uninvolved airway cells have an abnormal protein signature. Therapeutic intervention of the fibrotic uninvolved airway cells may therefore slow fibrosis progression.

## Supplementary Information

**Additional file 1: Figure S1.** Patient demographics. (A) Non-fibrotic and (B) fibrotic patient demographics. Lung function for fibrotic patients were taken as the last pulmonary function reading before transplant. **Figure S2.** Mucinous adenocarcinoma patient demographics. **Figure S3.** Elastin disorganization in the fibrotic honeycomb airway. 2 Non-fibrotic and 4 fibrotic specimens were stained for pentachrome or immunostained for elastin. Shown are representative images for (A) non-fibrotic airway, (B) fibrotic uninvolved airways, and (C) fibrotic honeycomb airways. Note that elastin fibres (black in color in the pentachrome) surround airways in the non-fibrotic airway and fibrotic uninvolved airways, but is disorganized in the honeycomb airways. **Figure S4.** The mucus in mucinous adenocarcinoma (MA). (A) A MA specimen was serially sectioned at 5 microns and stained with alcian blue/periodic acid Schiff’s (AB/PAS) stain or Hemetoxylin & Eosin (H&E). Mucus was laser microdissected for mass spectrometry analysis. Scale bar represented 100 microns. (B) A list showing the most abundant secretome-associated proteins found in the mucus of MA (n = 6 patients). (C) Dotplot visualization of the MA mucus using Reactome pathway enrichment.

**Additional file 2.** A list of detected airway proteins.

**Additional file 3.** Differentially expressed airway proteins.

**Additional file 4.** Significantly changed proteins in heatmap.

**Additional file 5.** A list of proteins detected in UIP and Mucinous Adenocarcinoma mucus.

**Additional file 6.** A list of mucus protein intensity levels.

**Additional file 7.** A list of mucus secretome associated proteins.

**Additional file 8.** Differentially expression mucus proteins in UIP versus Mucinous Adenocarcinoma.

## Data Availability

We have deposited the raw mass spectrometry data files to ProteomeXchange under the identifier of PD036465.
