# Supplementary material for: Morphologically intact airways in lung fibrosis have an abnormal proteome
Source: Respir Res. 2023 Apr 1;24:99. doi: 10.1186/s12931-023-02400-x (PMC10066954; doi:10.1186/s12931-023-02400-x)
Supplement: Supplementary file 1 — Additional file 1: Figure S1. Patient demographics. (A) Non-fibrotic and (B) fibrotic patient demographics. Lung function for fibrotic patients were taken as the last pulmonary function reading before transplant. Figure S2. Mucinous adenocarcinoma patient demographics. Figure S3. Elastin disorganization in the fibrotic honeycomb airway. 2 Non-fibrotic and 4 fibrotic specimens were stained for pentachrome or immunostained for elastin. Shown are representative images for (A) non-fibrotic airway, (B) fibrotic uninvolved airways, and (C) fibrotic honeycomb airways. Note that elastin fibres (black in color in the pentachrome) surround airways in the non-fibrotic airway and fibrotic uninvolved airways, but is disorganized in the honeycomb airways. Figure S4. The mucus in mucinous adenocarcinoma (MA). (A) A MA specimen was serially sectioned at 5 microns and stained with alcian blue/periodic acid Schiff’s (AB/PAS) stain or Hemetoxylin & Eosin (H&E). Mucus was laser microdissected for mass spectrometry analysis. Scale bar represented 100 microns. (B) A list showing the most abundant secretome-associated proteins found in the mucus of MA (n = 6 patients). (C) Dotplot visualization of the MA mucus using Reactome pathway enrichment. [file 12931_2023_2400_MOESM1_ESM.pptx]

## Slide 1
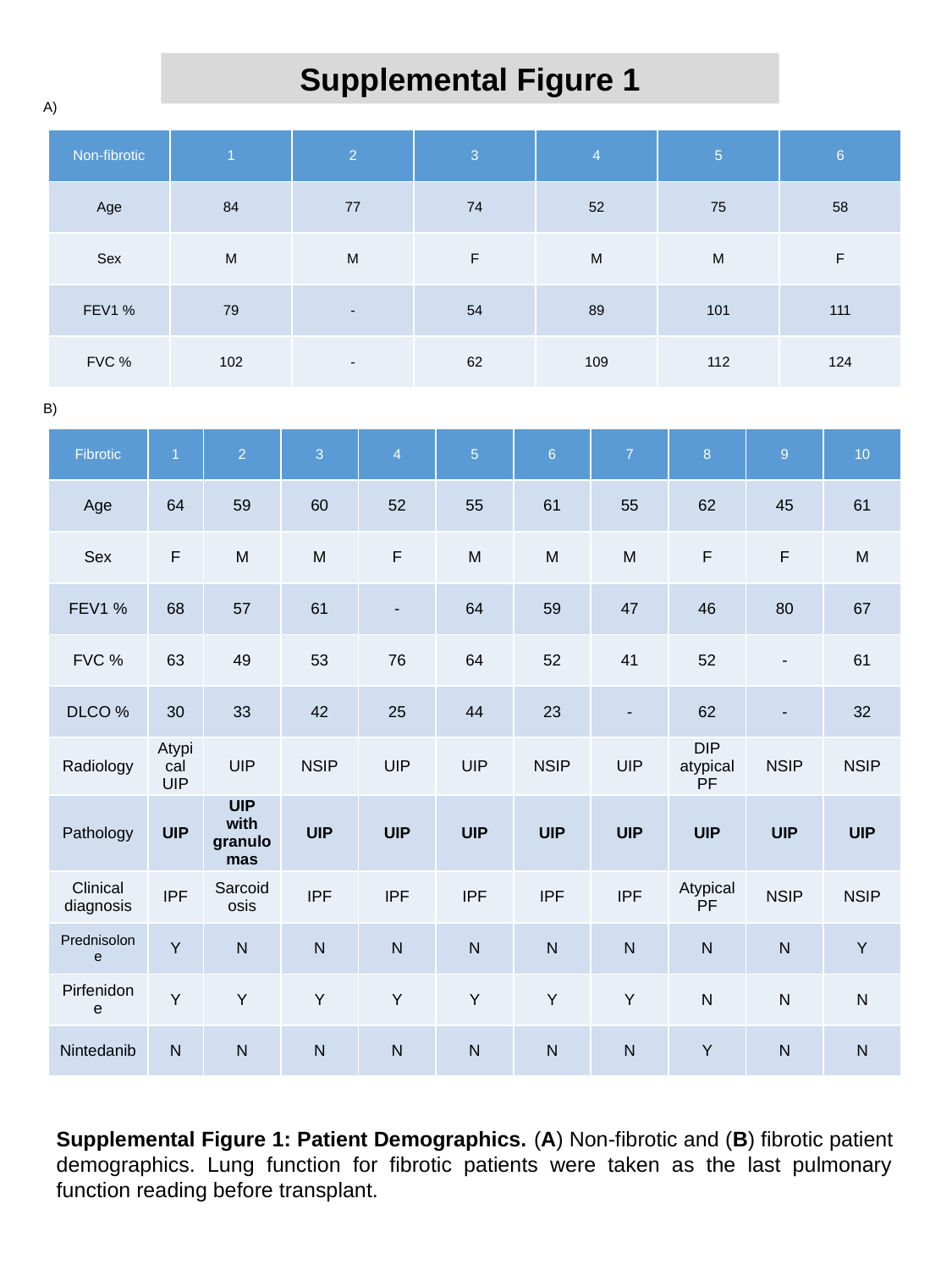

Supplemental Figure 1
A)
| Non-fibrotic | 1 | 2 | 3 | 4 | 5 | 6 |
| --- | --- | --- | --- | --- | --- | --- |
| Age | 84 | 77 | 74 | 52 | 75 | 58 |
| Sex | M | M | F | M | M | F |
| FEV1 % | 79 | - | 54 | 89 | 101 | 111 |
| FVC % | 102 | - | 62 | 109 | 112 | 124 |
B)
| Fibrotic | 1 | 2 | 3 | 4 | 5 | 6 | 7 | 8 | 9 | 10 |
| --- | --- | --- | --- | --- | --- | --- | --- | --- | --- | --- |
| Age | 64 | 59 | 60 | 52 | 55 | 61 | 55 | 62 | 45 | 61 |
| Sex | F | M | M | F | M | M | M | F | F | M |
| FEV1 % | 68 | 57 | 61 | - | 64 | 59 | 47 | 46 | 80 | 67 |
| FVC % | 63 | 49 | 53 | 76 | 64 | 52 | 41 | 52 | - | 61 |
| DLCO % | 30 | 33 | 42 | 25 | 44 | 23 | - | 62 | - | 32 |
| Radiology | Atypical UIP | UIP | NSIP | UIP | UIP | NSIP | UIP | DIP atypical PF | NSIP | NSIP |
| Pathology | UIP | UIP with granulomas | UIP | UIP | UIP | UIP | UIP | UIP | UIP | UIP |
| Clinical diagnosis | IPF | Sarcoidosis | IPF | IPF | IPF | IPF | IPF | Atypical PF | NSIP | NSIP |
| Prednisolone | Y | N | N | N | N | N | N | N | N | Y |
| Pirfenidone | Y | Y | Y | Y | Y | Y | Y | N | N | N |
| Nintedanib | N | N | N | N | N | N | N | Y | N | N |
Supplemental Figure 1: Patient Demographics. (A) Non-fibrotic and (B) fibrotic patient demographics. Lung function for fibrotic patients were taken as the last pulmonary function reading before transplant.

## Slide 2
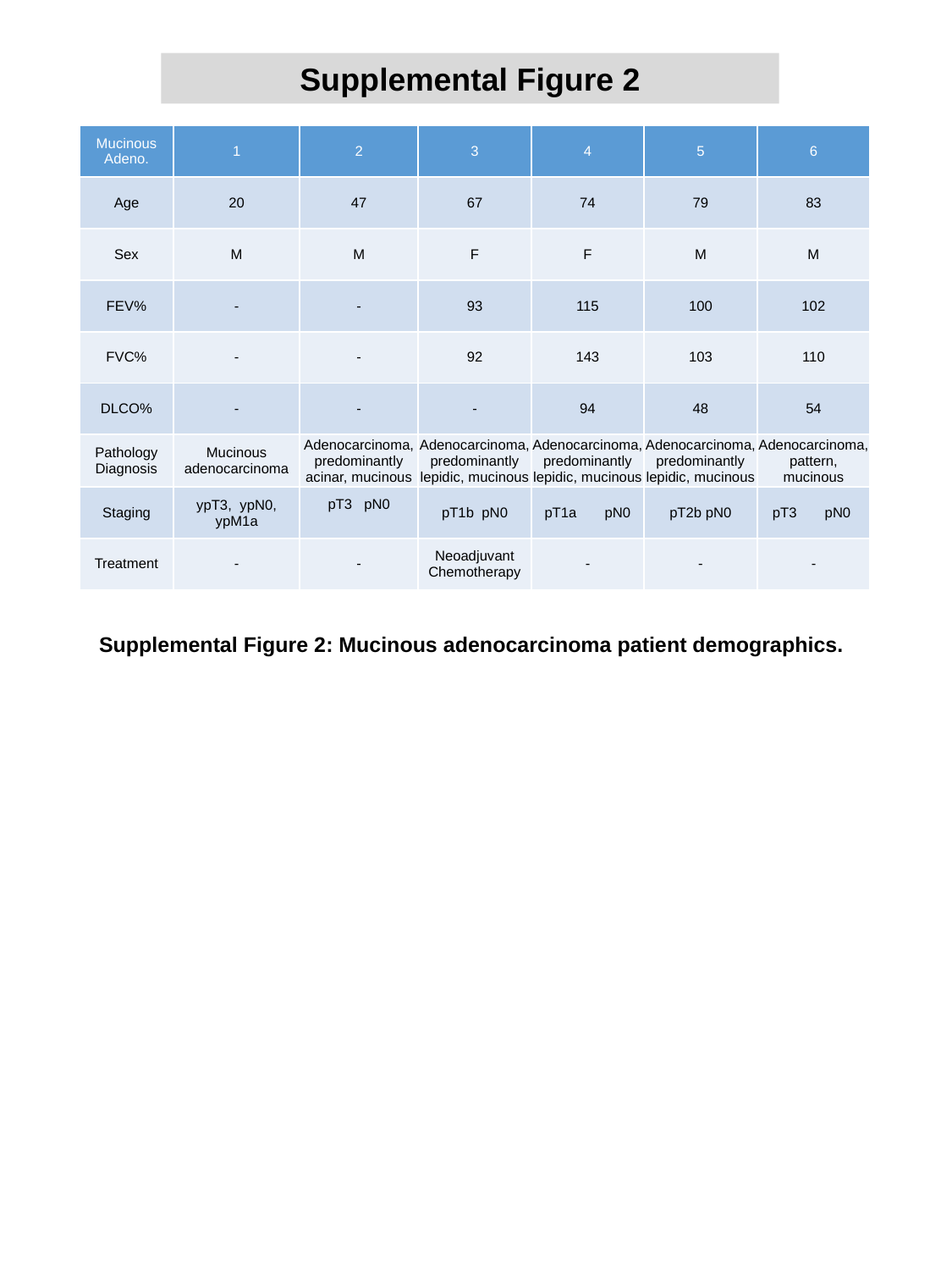

Supplemental Figure 2
| Mucinous Adeno. | 1 | 2 | 3 | 4 | 5 | 6 |
| --- | --- | --- | --- | --- | --- | --- |
| Age | 20 | 47 | 67 | 74 | 79 | 83 |
| Sex | M | M | F | F | M | M |
| FEV% | - | - | 93 | 115 | 100 | 102 |
| FVC% | - | - | 92 | 143 | 103 | 110 |
| DLCO% | - | - | - | 94 | 48 | 54 |
| Pathology Diagnosis | Mucinous adenocarcinoma | Adenocarcinoma, predominantly acinar, mucinous | Adenocarcinoma, predominantly lepidic, mucinous | Adenocarcinoma, predominantly lepidic, mucinous | Adenocarcinoma, predominantly lepidic, mucinous | Adenocarcinoma, pattern, mucinous |
| Staging | ypT3, ypN0, ypM1a | pT3 pN0 | pT1b pN0 | pT1a pN0 | pT2b pN0 | pT3 pN0 |
| Treatment | - | - | Neoadjuvant Chemotherapy | - | - | - |
Supplemental Figure 2: Mucinous adenocarcinoma patient demographics.

## Slide 3
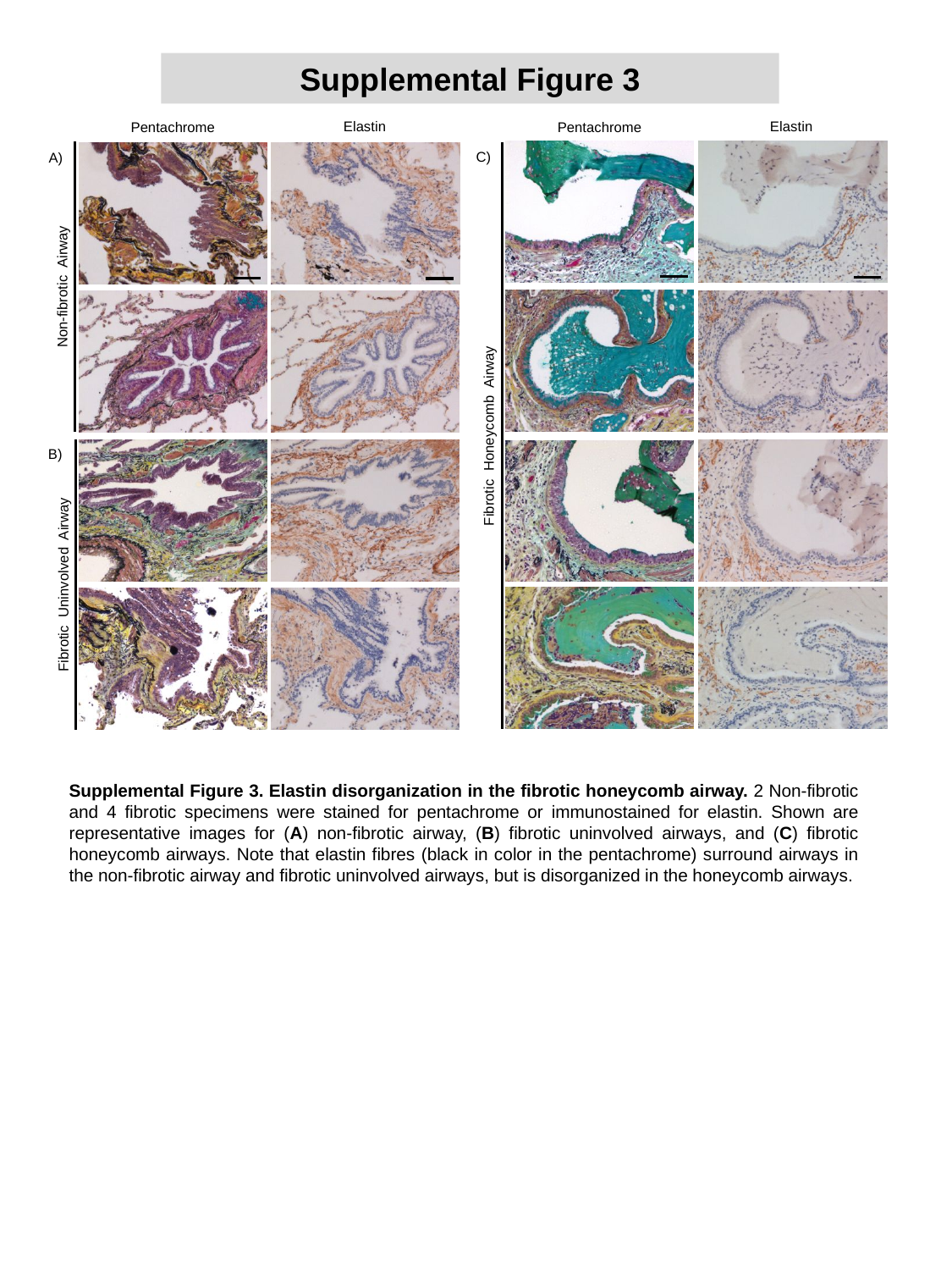

Supplemental Figure 3
Elastin
Elastin
Pentachrome
Pentachrome
C)
A)
Non-fibrotic Airway
Fibrotic Honeycomb Airway
B)
Fibrotic Uninvolved Airway
Supplemental Figure 3. Elastin disorganization in the fibrotic honeycomb airway. 2 Non-fibrotic and 4 fibrotic specimens were stained for pentachrome or immunostained for elastin. Shown are representative images for (A) non-fibrotic airway, (B) fibrotic uninvolved airways, and (C) fibrotic honeycomb airways. Note that elastin fibres (black in color in the pentachrome) surround airways in the non-fibrotic airway and fibrotic uninvolved airways, but is disorganized in the honeycomb airways.

## Slide 4
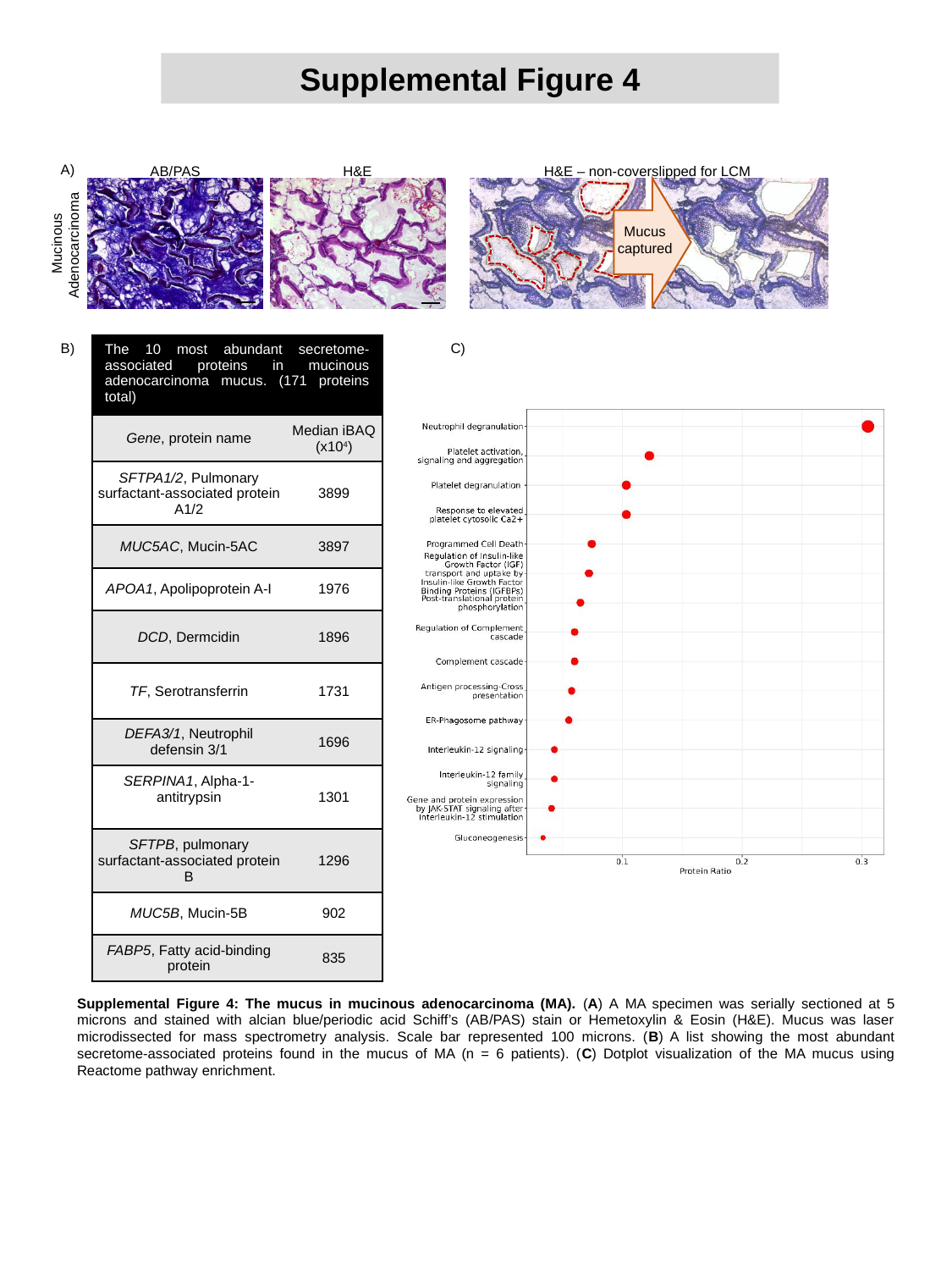

Supplemental Figure 4
A)
H&E – non-coverslipped for LCM
AB/PAS
H&E
Mucus captured
Mucinous Adenocarcinoma
B)
C)
| The 10 most abundant secretome-associated proteins in mucinous adenocarcinoma mucus. (171 proteins total) | |
| --- | --- |
| Gene, protein name | Median iBAQ (x104) |
| SFTPA1/2, Pulmonary surfactant-associated protein A1/2 | 3899 |
| MUC5AC, Mucin-5AC | 3897 |
| APOA1, Apolipoprotein A-I | 1976 |
| DCD, Dermcidin | 1896 |
| TF, Serotransferrin | 1731 |
| DEFA3/1, Neutrophil defensin 3/1 | 1696 |
| SERPINA1, Alpha-1-antitrypsin | 1301 |
| SFTPB, pulmonary surfactant-associated protein B | 1296 |
| MUC5B, Mucin-5B | 902 |
| FABP5, Fatty acid-binding protein | 835 |
Supplemental Figure 4: The mucus in mucinous adenocarcinoma (MA). (A) A MA specimen was serially sectioned at 5 microns and stained with alcian blue/periodic acid Schiff’s (AB/PAS) stain or Hemetoxylin & Eosin (H&E). Mucus was laser microdissected for mass spectrometry analysis. Scale bar represented 100 microns. (B) A list showing the most abundant secretome-associated proteins found in the mucus of MA (n = 6 patients). (C) Dotplot visualization of the MA mucus using Reactome pathway enrichment.
